# Fabrication of Troponin I Biosensor Composed of Multi-Functional DNA Structure/Au Nanocrystal Using Electrochemical and Localized Surface Plasmon Resonance Dual-Detection Method

**DOI:** 10.3390/nano9071000

**Published:** 2019-07-11

**Authors:** Taek Lee, Jinmyeong Kim, Inho Nam, Yeonju Lee, Ha Eun Kim, Hiesang Sohn, Seong-Eun Kim, Jinho Yoon, Sang Woo Seo, Min-Ho Lee, Chulhwan Park

**Affiliations:** 1 Department of Chemical Engineering, Kwangwoon University, Wolgye-dong, Nowon-gu, Seoul 01899, Korea; 2 Department of Chemical Engineering and Materials Science, Chung-Ang University, Heukseok-dong, Dongjak-gu, Seoul 06974, Korea; 3 Human IT Convergence Research Center, Korea Electronics Technology Institute, Seongnam-si, Gyeonggi-do 13509, Korea; 4 Department of Chemical and Biomolecular Engineering, Sogang University, 35 Baekbeom-ro, Mapo-gu, Seoul 04107, Korea; 5 School of Chemical and Biological Engineering, Institute of Chemical Process, Seoul National University, Seoul 08826, Korea; 6 School of Integrative Engineering Chung-Ang University, Heukseok-dong, Dongjak-gu, Seoul 06974, Korea

**Keywords:** dual-mode biosensor, cardiac troponin I biosensor, multi-functional DNA structure, electrochemical biosensor, LSPR, EC-LSPR

## Abstract

In the present study, we fabricated a dual-mode cardiac troponin I (cTnI) biosensor comprised of multi-functional DNA (MF-DNA) on Au nanocrystal (AuNC) using an electrochemical method (EC) and a localized surface plasmon resonance (LSPR) method. To construct a cTnI bioprobe, a DNA 3 way-junction (3WJ) was prepared to introduce multi-functionality. Each DNA 3WJ arm was modified to possess a recognition region (Troponin I detection aptamer), an EC-LSPR signal generation region (methylene blue: MB), and an anchoring region (Thiol group), respectively. After an annealing step, the multi-functional DNA 3WJ was assembled, and its configuration was confirmed by Native-TBM PAGE for subsequent use in biosensor construction. cTnI was also expressed and purified for use in biosensor experiments. To construct an EC-LSPR dual-mode biosensor, AuNCs were prepared on an indium-tin-oxide (ITO) substrate using an electrodeposition method. The prepared multi-functional (MF)-DNA was then immobilized onto AuNCs by covalent bonding. Field emission scanning electron microscope (FE-SEM) and atomic force microscopy (AFM) were used to analyze the surface morphology. LSPR and electrochemical impedance spectroscopy (EIS) experiments were performed to confirm the binding between the target and the bioprobe. The results indicated that cTnI could be effectively detected in the buffer solution and in diluted-human serum. Based on the results of these experiments, the loss on drying (LOD) was determined to be 1.0 pM in HEPES solution and 1.0 pM in 10% diluted human serum. Additionally, the selectivity assay was successfully tested using a number of different proteins. Taken together, the results of our study indicate that the proposed dual-mode biosensor is applicable for use in field-ready cTnI diagnosis systems for emergency situations.

## 1. Introduction

As the obesity rate and the overall age of the population are both increasing worldwide, concerns of higher incidences of cardiovascular diseases (CVDs) are gradually growing [1]. CVDs are regarded as one of the major causes of human death in the world population [2]. Among CVDs, acute myocardial infarction (AMI) in the aging society is one of the most serious diseases due to its high mortality rate and low recovery rate [3,4]. AMI occurs when the heart muscle experiences aberrant blood flow fluctuation or coronary artery blockage. Upon experiencing AMI, the patient should be treated and transported to the hospital within 1 h for the highest chance of recovery. After 1 h with lack of proper treatment, the patient can experience multi-organ failure that can ultimately lead to death or loss of brain function. Given this, rapid and appropriate treatment of AMI is critical for patient survival [5].

Several biomarkers are capable of detecting AMI, and these include cardiac troponin (C, T and I), myoglobin (MB), creatine kinase-MB, and MB isozymes [6,7]. Among these, the Troponin complex is comprised of regulatory proteins that are related to cardiac muscle contraction, and this complex contains three subunits identified as specifically troponin C, T, and I. Troponin C, T, and I are unique proteins found within the heart muscle cells that when detected in the blood can be powerful markers for AMI determination [8]. In particular, cardiac troponin I (cTnI) serves as a useful biomarker for the determination of AMI occurrence and for the time that has elapsed since the AMI due to the positive relationship between cTnI levels and AMI symptoms. The molecular weight of cTnI is 23.9 kDa, and the theoretical pI of cTnI is 9.05 [9]. Given this, this complex can be easily expressed by conventional DNA recombinant technology for the purposes of treatment and diagnosis [10].

Currently, several types of cTnI detection methods have been developed to detect cTnI sensitively and selectively [9,11]. The field effect transistor-based detection method [12,13], electrochemical detection method [10,11,14], fluorescence method [15,16], surface-enhanced Raman spectroscopy (SERS) method [17], colorimetric method [18], and surface plasmon resonance (SPR) spectroscopy-based detection method [11,19,20] have all been developed for use as cTnI biosensors. Those biosensors exhibit different pros and cons concerning sample preparation, operation time, labeling, sensitivity, cost, and portability. For cTnI detection, rapid detection time, ultrasensitivity (less than ~1 pM), and portability are all required, as these are all critical for effective use in emergent situations that may arise within the home or the ambulance. To meet these requirements, the electrochemical method EC-based and LSPR-based detection methods are suitable due to their simplicity, ease of operation, and low detection limit. However, the EC-based detection method requires a labeling process and a redox mediator, while the localized surface plasmon resonance LSPR-based detection method requires a well-ordered nanostructure and has a low reproducibility.

**Figure 1:**
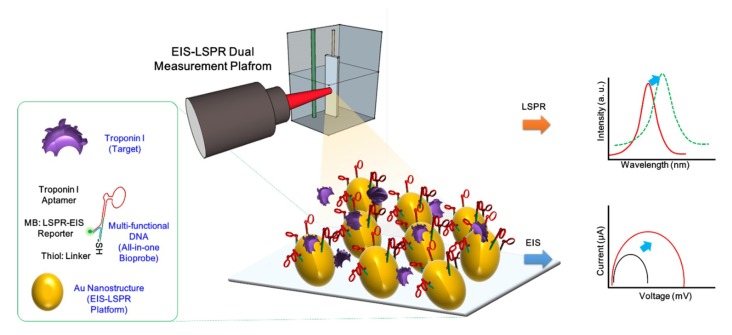
Schematic image of the fabricated electrochemical method (EC) localized surface plasmon resonance (LSPR) biosensor for cardiac troponin I (cTnI) detection.

To solve these problems, for the first time, we fabricated a cTnI dual detection biosensor composed of a multi-functional DNA 3 way-junction (3WJ)-based bioprobe immobilized onto an Au nanocluster (AuNC)-modified ITO substrate using EC-LSPR methods. To harness the required multi-functionality, nucleic acid possessed various advantages such as ease of tailoring and the ability to regulate their length, compared to antibodies. However, the multi-functionality of conventional double strand-based nucleic acid is limited due to the number of arms. To simultaneously perform three functions, DNA 3WJ is suitable for target binding, signal reporting, and anchoring to substrate [14,21,22]. The cTnI aptamer was introduced at the end of the DNA 3WJa fragment to detect cTnI. For EC-LSPR signal generation, methylene blue (MB) was incorporated into the DNA 3WJb fragment. Additionally, it has been reported that fluorescence dye (FAM) or redox dye (methylene blue) can enhance the plasmonic effects that may be increased in the LSPR band [23,24]. MB-tagged DNA 3WJb may enhance the sensitivity. Finally, the thiol-modified DNA 3WJc fragment was introduced to immobilize the substrate without the use of specific linker material. Apt/MB/SH-3WJ assembly was achieved by an annealing process, and the result was confirmed by the TBM Native-PAGE experiment. The electrodeposition method was then used to add the AuNC onto the ITO substrate that can be directly applied to EC-LSPR detection [25]. This simple nanostructure provides a higher surface coverage that facilitates increased immobilization time for greater target-bioprobe binding. Additionally, this process does not require complicated biosensor fabrication methods, ultimately reducing biosensor manufacture time. Clinical testing was conducted using an artificial serum sample. The analytical performances were evaluated by EC-LSPR experiments, and (Figure 1) shows the schematic diagram of the cTnI detection system.

## 2. Experimental Details

### 2.1. Materials

To synthesize AuNC on the ITO substrate, Hydrogen tetrachloroaurate(III) trihydrate (HAuCl_4_·3H_2_O), Triton-X solution, ammonium sulfate, potassium citrate tribasic monohydrate, and sodium hydroxide were purchased from Sigma-Aldrich (Saint Louis, MO, USA). The three fragments of DNA 3WJ were provided by Bioneer (Daejon, South Korea). The sequence of the cTnI aptamer was utilized according to the method provided by the Ban group [10]. To have the multi-functionality, the DNA 3WJ was connected to the Tro6 aptamer. The sequence of Tro6 aptamer-tagged 3WJ-a (Tro6/3WJa) was 5′-CGC ATG CCA AAC GTT GCC TCA TAG TTC CCT CCC CGT GTC CTT GCC ATG TGT ATG TGG G-3’. The methylene blue-tagged 3WJ-b (MB/3WJb) sequence was MB-5’-TTT GGG TAG GGC GGG TTG GG C CCA CAT ACT TTG TTG ATC C-3’, and the Thiol-tagged 3WJ-c (SH/3WJc) sequence was SH-5’-GGA TCA ATC ATG GCA A-3′. For obtaining the cTnI, To prepare cTnI plasmid, the sequence of the forward primer was 5′-GCG GAT CCA TGG CGG ATG GGA GCA G-3′, and the sequence of reverse primer was 5′-GCA AGC TTT CAG CTC TCA AAC TTT TTC TTG CGG-3′. The detailed sequence information of fabricated DNA 3WJ was described in our previous work [14]. To dilute all oligonucleotides, nuclease free water was used. To evaluate the fabricated biosensor in clinical conditions, human serum from human male AB plasma (USA origin) was purchased from Sigma-Aldrich (Saint Louis, MO, USA). For the selectivity test, bovine serum albumin (BSA), cytochrome c (Cyt c), hemoglobin (Hemo) and myoglobin (Myo) were purchased from Sigma-Aldrich (Saint Louis, MO, USA). Hydrogen peroxide (30%), and dimethylamine, and silver nitrate were purchased from Daejung Fine Chemicals (Daejon, South Korea). The dimethylamine was utilized as the buffer for electrochemical impedance spectroscopy (EIS) experiment. Hydrogen tetrachloroaurate (III) hydrate was purchased from Kojima Chemicals Co. (Sayama, Japan). 

### 2.2. Construction of cTnI Plasmid, and Expression and Purification of cTnI

The cTnI gene fragments were amplified from HEK293 cDNA by polymerase chain reaction (PCR) using purchased oligonucleotide primers with *Bam*HI/*Hind*III and *Eco*RI/*Xho*I restriction enzyme sites (*cTnI* primers:5′-GCGGATCCATGGCGGATGGGAGCAG-3′; 5′-GCAAGCTTTCAGCTCTCAA ACTTTTTCTTGCGG-3′). The PCR was performed with the conditions previously described by Jo et al. [10]. The gene encoding cTnI was amplified from the HEK293 cDNA using polymerase chain reaction (PCR), and the cTnI gene was optimized by Genscript (USA) as shown in the Supplementary Materials. The amplified cTnI gene was inserted into pET28a expression vectors containing a (His)_6_-tag at the N-terminal. An *E. coli* strain (DH5a) was used as the host for subcloning. Standard subcloning techniques were performed in this work. The forward primer was designed to have a *Bam*HI restriction enzyme site, and the reverse primer was designed to contain a *Hind*III restriction enzyme site. The PCR product was isolated using a DNA purification kit (Qiazen, Hilden, Germany), and digested with two restriction enzymes for *Bam*HI and *Hind*III (New England Biolabs, Ipswich, MA, USA). The digested DNA fragments were ligated into a pET-28a vector (Merck, Kenilworth, NJ, USA) that was previously digested with *Bam*HI and *Hind*III using a ligation kit (TaKaRa, Shiga, Japan). To obtain the cTnI protein, the pET28a vector encoding cTnI gene was transformed into *E. coli* BL21 (DE3) [10,26]. The cTnI-expressing cells were resuspended in lysis buffer (20 mM Tris, pH 8.0, 500 mM NaCl, 0.5 mM β-mercaptoethanol, and 0.1% Tween-20) and then disrupted by sonication on ice. Soluble cTnI protein in the supernatant was purified via a Ni-NTA-packed column (Qiazen). Then, the collected cTnI was concentrated using MWCO 3k Amicon Ultra centrifugal filter (Millipore, USA) with 10 mM HEPES buffer. The purified cTnI protein was confirmed by 12% SDS-PAGE (Figure S1).

### 2.3. Fabrication of AuNC on ITO Substrate

AuNC was electrochemically synthesized using a potentiostat (Versastat3 potentiostat, Princeton Applied Research, Tulsa, OR, USA). For the three-electrode setup, an ITO-coated glass substrate (10 Ω, Nanofab Center, South Korea) was used as a working electrode, a platinum wire was used as the counter electrode, and Ag/AgCl served as the reference electrode. ITO-coated glass substrates were cleaned by sonication for 30 min using 2% Triton X-100 solution, deionized water (DIW), and ethanol. AuNC was electrochemically deposited onto ITO substrates (30 mm × 10 mm) using 5 mM HAuCl_4_, 0.5 mM ammonium sulfate, and 2 mM potassium citrate tribasic monohydrate. The potential was applied at −1.3 V (vs. Ag/AgCl) for a fixed time (120 s), and the deposition was performed at 25 °C. After the deposition, the ITO electrode was removed from the electrochemical cell, rinsed with DIW, and air-dried for further use [25]. Several electrodeposition experiments were carried out to determine the optimal conditions (Figure S2). To observe the surface topography of AuNC synthesized on ITO-substrate, field-effect scanning electron microscopy (FE-SEM) (Auriga, Carl Zeiss, Oberkochen, Germany) was utilized.

### 2.4. Assembly of Multi-Functional DNA 3WJ

To assemble multi-functional DNA structure, DNA 3WJ fragments were modified with a functional group. Tro6/3WJa (5′-CGC ATG CCA AAC GTT GCC TCA TAG TTC CCT CCC CGT GTC CTT GCC ATG TGT ATG TGG G-3′) was introduced. For the generation of simultaneous plasmonic signal and electrochemical signal, MB-tagged DNA 3WJb was added (MB/3WJb, 5′-CCC ACA TAC TTT GTT GAT CC-3′). Finally, thiol-modified DNA 3WJc was prepared to allow for anchoring of the AuNC directly (SH/3WJc, SH-5′-GGA TCA ATC ATG GCA A-3′). All above DNA fragments were all chemically synthesized by Bioneer (Daejon, South Korea). The AIapt/MB/SH-3WJ was finally assembled by annealing three corresponding DNA strands at an equal molar ratio in TMS buffer (40 mM Tris-HCl, 10 mM MgCl_2_, 100 mM NaCl) by heating at 80 °C for 5 min, followed by slowly cooling down to 4 °C at a rate of 2 °C/min on a T100™ Thermal Cycler (Bio-rad, Hercules, CA, USA). The assembly of prepared DNA bioprobes was confirmed by our previous study [14,26].

### 2.5. Fabrication and Investigation of Multi-Functional DNA 3WJ/AuNC Heterolayer

To immobilize a multi-functional DNA 3WJ/AuNC onto an ITO substrate, the fabricated electrode was treated with acetone solution and then sonicated for 5 min, washed with ethanol and deionized water, and dried under N_2_ gas to remove any residues. Then, 3 μL of 1 μM multi-functional DNA was added onto the AuNC surface for 3 h. The thiol group of the multi-functional DNA molecule was conjugated onto the AuNC surface by covalent bonding. The excess biomolecule was removed by DIW and N_2_ gas stream [27].

The AuNC-modified ITO substrates were analyzed by FE-SEM (Auriga, Carl Zeiss, Oberkochen, Germany), and the biofilm formation was investigated by AFM (Digital Instruments, USA). The bare ITO substrate, AuNC-modified ITO, DNA 3WJ self-assembled onto AuNC-modified ITO substrate, and the HA protein/DNA 3WJ/AuNC-modified ITO substrates were all characterized for comparison. Prior to scanning the sample, all the conditions such as the set point current, integrated gain, and proportional gain were optimized to the force between the tip and the substrate surface [25]. The AFM image size was 800 nm × 800 nm. To observe the vertical surface properties, the surface roughness and sectional analysis of each image was obtained by Nanoscope software. The analytical parameters for the fabricated heterolayer surface investigation were the root mean square (RMS), roughness (R_q_), the roughness max (R_max_), the roughness average (R_a_) and the vertical distance.

### 2.6. Optical Analysis of cTnI Detection by Localized Surface Plasmon Resonance Method

To optimize the immobilization condition of the Tro6/MB/SH-DNA 3WJ, the LSPR method was carried out. The cTnI protein binding to the Tro6/MB/SH-DNA 3WJ was analyzed by the peak shift of LSPR in the UV–Vis spectrum resulting from the changes in the local refractive index induced by the target-aptamer reaction. The local refractive index changes by target-aptamer interaction at a given wavelength took place because of the extension of light absorption of the biofilm on the AuNC-modified ITO electrode. All absorption spectra were observed by monitoring UV–Vis spectral changes in the transmission mode using a JASCO V-530 UV-spectrometer [25].

### 2.7. Electrochemical Analysis of cTnI Detection by EIS

To perform cyclic voltammetry (CV) and electrochemical impedance spectroscopy (EIS) experiments, a customized-three-electrode system setup was utilized. The DNA 3WJ/AuNC-ITO electrode served as the working electrode. Pt electrodes and Ag/AgCl electrodes were mounted on the top of the fabricated electrode used as a counter electrode and reference electrode, respectively. CV and EIS were performed in 0.5 M of dimethylamine. The potential for CV was cycled from −0.9 V to 0.9 V at a 0.05 V/s scan rate. The EIS analysis was set to measure the electron transfer resistance in the frequency ranging from 1 Hz to 100 kHz under a potential of 0.2 V. The potential was decided as a redox potential that was determined by the initial CV analysis (Figure S3). The collected data were fitted using the origin graphing software for calculating the values of electron transfer resistance (R_et_). The electrode response can be expressed as (R_i_ − R_o_)/R_o_, where R_o_ depicted the electron transfer resistance of the fully modified electrode obtained from the dimethylamine buffer prior to aptamer immobilization and Ri represented the electron transfer resistance of the fully modified electrode obtained from the specific concentration of the aptamer-target in the same buffer. All electrochemical measurements were performed using a Versastat3 potentiostat (Princeton Applied Research, Tulsa, OR, USA) [28].

## 3. Results and Discussion

### 3.1. Investigation of Fabricated cTnI/Multi-Functional DNA on AuNC-Modified ITO Substrate

**Figure 2:**
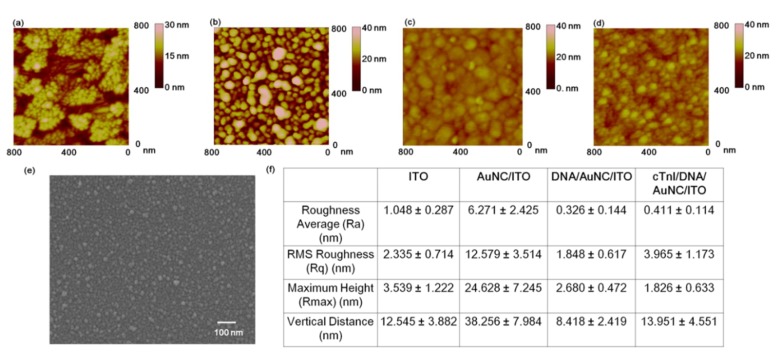
AFM data for the surface morphology investigation, (**a**) Indium-tin-oxide (ITO) substrate, (**b**) AU nanocluster (AuNC)-deposited ITO substrate, (**c**) DNA 3 way junction (3WJ)-modified AuNC-deposited ITO substrate, (**d**) cTnI/DNA 3WJ/AuNC-modified ITO substrate, (**e**) Surface roughness analysis values of the ITO, AuNC on ITO, DNA 3WJ-modified AuNC-ITO substrate and cTnI on DNA 3WJ/AuNC-ITO substrate, (**f**) Surface roughness analysis of the ITO, AuNC/ITO, DNA 3WJ/AuNC/ITO and HA protein/DNA 3WJ/AuNC/ITO.

The AFM experiments and surface roughness analysis were performed to confirm the formation of AuNC onto the ITO substrate and the immobilization process of cTnI onto the multi-functional DNA 3WJ on AuNC-modified substrate, respectively. (Figure 2a) indicates the topography of cleaned ITO substrate by AFM. A grain of 20–30 nm was observed, and the R_a_ value was 1.048 ± 0.287 nm, the RMS roughness (R_q_) was 2.335 ± 0.714 nm, the R_max_ was 3.539 ± 1.222 nm, and the vertical distance (VD) between the surface and the top of the ITO was 12.545 ± 3.882 nm. After AuNC formation on the ITO substrate by the electrodeposition process, the grain size was increased to 60 to 90 nm compared to ITO substrate, which is similar to that observed in the FE-SEM image of AuNC-modified ITO substrate (Figure 2b). In regard to the AuNC, the R_a_ value was 6.271 ± 2.425 nm, and the RMS roughness (R_q_) was 12.579 ± 3.514 nm, the R_max_ was 24.628 ± 7.245 nm, and the vertical distance (VD) between ITO substrate and the top of AuNC was 38.256 ± 7.984 nm. When the multi-functional DNA 3WJ was immobilized (Figure 2c), the AuNC grains were covered with DNA molecules that resulted in a dimmer surface compared to that of the AuNC-modified ITO substrate surface. For the DNA 3WJ surface roughness values, the R_a_, R_q_, R_max_, and VD were 0.326 ± 0.144 nm, 1.848 ± 0.617 nm, 2.680 ± 0.482 nm and 8.418 ± 2.419 nm, respectively. These decreased values may be a result of the dim surface topography that occurred when the DNA 3WJ was introduced. (Figure 2d) presents the surface morphology of cTnI reacted with the DNA 3WJ/AuNC-modified ITO substrate. Compared to the DNA 3WJ/AuNC-modified substrate, the sphere lumps (30–40 nm) formed on the surface may indicate the formation of cTnI onto the bioprobe. The R_a_, R_q_, R_max_, and VD were 0.411 ± 0.114 nm, 3.965 ± 1.173 nm, 1.826 ± 0.633 nm, and 13.951 ± 4.551 nm, respectively. Additionally, FE-SEM analysis provided further details concerning the fabricated AuNC-modified ITO substrate (Figure 2e). The surface roughness values of the ITO, AuNC-deposited ITO, DNA 3WJ on AuNC, and the cTnI reacted with DNA 3WJ on AuNC are listed in (Figure 2f). Based on the AFM analysis, the fabricated DNA 3WJ/AuNC-modified substrate was suitable for cTnI biosensor application.

### 3.2. Optical and Electrochemical Properties of Fabricated Biosensor

**Figure 3:**
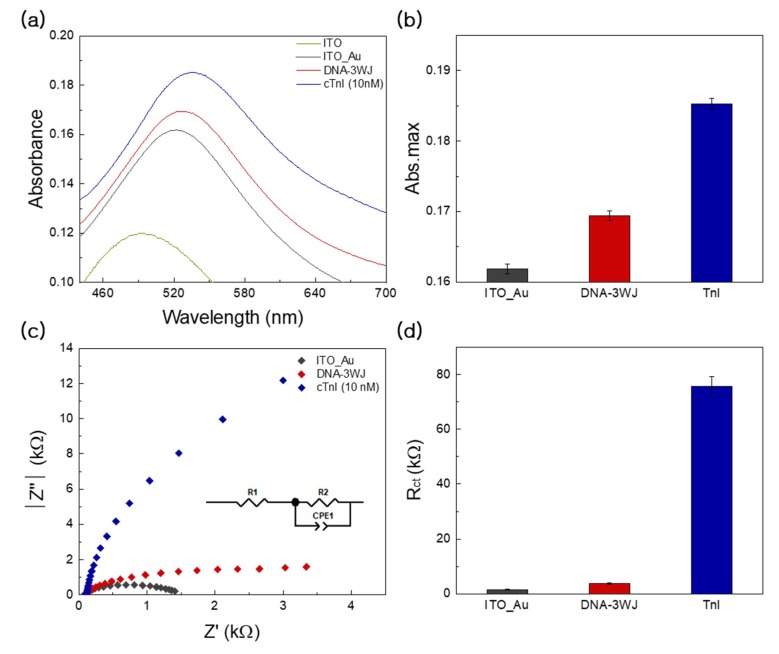
(**a**) Optical characteristics wavelength between 440 to 680 nm of the cTnI/DNA 3WJ/AuNC on ITO substrate (black line), DNA 3WJ layer (red line), cTnI reacted with DNA 3WJ (blue line), respectively. (**b**) Change in absorbance peak based on immobilization of AuNC, DNA 3WJ, cTnI protein layers, respectively. (**c**) Electrochemical impedance spectra of cTnI/DNA 3WJ/AuNC on ITO substrate (black line), DNA 3WJ layer rRed line), cTnI reacted with DNA 3WJ (blue line), respectively. (**d**) Change in R^2^ immobilization of AuNC, DNA 3WJ, cTnI protein layers, respectively. Error bar represents relative standard deviation of eight independent experiments.

After the fabrication of AuNC onto ITO substrate, the LSPR band was obtained in the visible range at 552 nm (Figure 3, black color). Then, the immobilization of multi-functional DNA 3WJ altered the LSPR intensity increment and caused a red shift in the wavelength (527 nm, Figure 3, red color). After the addition of cTnI to the multi-functional DNA 3WJ/AuNC-modified electrode, the specific binding event between cTnI and multi-functional DNA 3WJ may be increased at specific absorbance intensity increments of 536 nm (Figure 3, blue color). (Figure 3a) depicts the optical characteristics of the cTnI/MF-DNA-modified Au NC on ITO substrate. (Figure 3b) presents the graph representing the LSPR peak of the fabricated cTnI/MF-DNA on the AuNC electrode. The results indicated a band-shift well after biofilm fabrication on the nanostructure, and those results were confirmed by EIS experiments. (Figure 3c) indicates the Nyquist plots of AuNC on ITO, the immobilized DNA 3WJ on the AuNC/ITO substrate, and cTnI/MF-DNA-modified Au NC/ITO substrate.

The analysis of the EIS spectrum was performed by a simple Randles circuit as an equivalent circuit. In the equivalent circuit (inset of (Figure 3c)), *R*_bulk_ is the bulk resistance of the electrolyte, and *R*_ct_ is the charge-transfer resistance for the redox reaction. The kinetics parameters extracted from the fitting corroborated the reaction path of the three electrode systems. The *R*_bulk_ values were similar for all the electrodes; however, *R*_ct_ significantly varied. Therefore, it should be noted that the conducting paths are decreased by the sequential addition of materials, indicating that the sequential reaction is well fabricated by our scheme. In particular, upon addition of Tni, *R*^2^ is sharply increased compared to that observed after addition of DNA 3WJ, indicating that the EIS analysis may be highly sensitive to Tni concentration. In conclusion, the prepared DNA 3WJ/AuNC-modified electrode can be used as a cTnI detection biosensor based on the aptamer-target recognition reactions.

### 3.3. Detection Performance and Clinical Test Using LSPR

**Figure 4:**
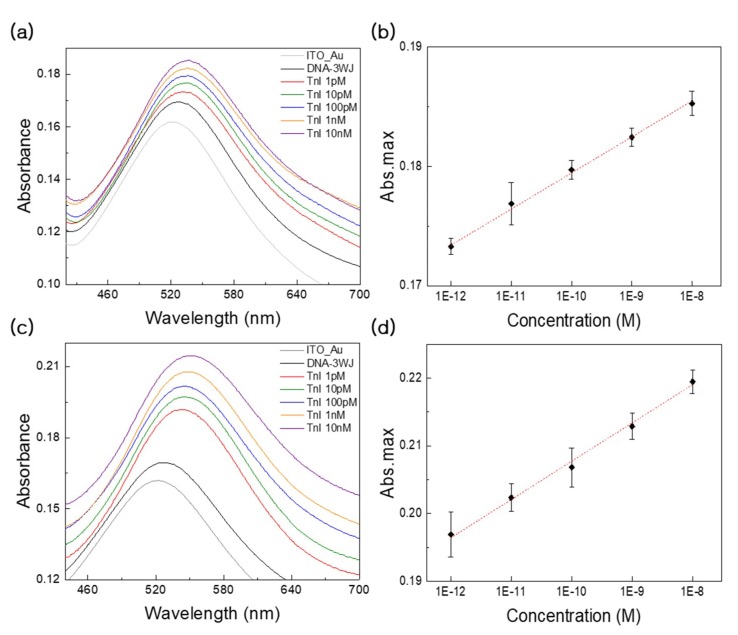
(**a**) Absorbance peak change at wavelength around 550 nm LSPR absorbance strength changes with different cTnI concentrations in PBS buffer, respectively. (**b**) Linear curves of the different concentration of cTnIs range from 1 pM to 10 nM. The detection limit of LSPR based biosensor for cTnI was 1 pM. (**c**) Absorbance peak change at wavelength around 550 nm LSPR absorbance strength changes with different cTnI concentrations in 10% diluted human serum, respectively. (**d**) Linear curves of the different concentration of cTnIs range from 1 pM to 10 nM. The detection limit of LSPR based biosensor for cTnI was 1 pM. Error bar represents relative standard deviation of eight independent experiments.

LSPR measurements were performed on the prepared DNA 3WJ/AuNC-modified electrode after that addition of cTnI. To analyze the cTnI detection performance in the context of loss on drying (LOD), dynamic range, and selectivity, LSPR experiments were conducted on eight samples. Additionally, the fabricated LSPR cTnI biosensor was tested with cTnI in diluted human serum to determine clinical suitability. After the washing step, the cTnI was reacted with the DNA 3WJ/AuNC-modified ITO electrode for 2 h at RT. The obtained LSPR bands corresponded to the serially diluted cTnI concentrations (0 pM, 1 pM, 10 pM, 100 pM, 1nM, and 10 nM). The various concentration changes in cTnI dissolved in PBS buffer were observed by the absorbance changes as refractive index (RI) changes on the AuNC surface. Increment of absorbance intensity values and redshifts in the wavelengths of LSPR bands were monitored when the cTnI concentration was increased (Figure 4a). These phenomena may be explained by the number of aptamer-target recognition events occurring at the surface that result in LSPR bands shifts. The incrementally increasing LSPR peak intensities corresponded well with increasing substrate concentrations.

Figure 4b illustrates the linear curve relationship between the absorbance and the cTnI concentrations. In this range, a positive linear relationship was obtained between the target concentration and the absorbance. The LOD of the fabricated DNA 3WJ/AuNC-modified electrode was estimated at 110 fM in PBS buffer through error bar and slopes of (Figure 4b). The dynamic range obtained in the concentration range of 1.0 pM to 10 nM exhibited linearity with increasing cTnI concentration with a correlation coefficient of 0.9981. The linear equation was y = 0.003x + 0.2096, and the R² = 0.9981. Additionally, for the clinical test, LOD tests were performed using the 10% diluted human serum. (Figure 4c,d) indicate the LSPR bands that were obtained using the serially diluted cTnI concentrations (0 pM, 1 pM, 10 pM, 100 pM, 1nM and 10 nM) and the linear curve. The LOD of the fabricated DNA 3WJ/AuNC-modified electrode was determined 840 fM in diluted serum through error bar and slopes of (Figure 4d). The linear equation was y = 0.0057x + 0.2593, and the R² = 0.9956. Based on these results, the LSPR analysis revealed a high relationship between the concentration of cTnI and the LSPR band shift observed in the dynamic range (1 pM, 10 pM, 100 pM, 1nM, and 10 nM).

### 3.4. Detection Performance and Clinical Testing Using EIS

**Figure 5:**
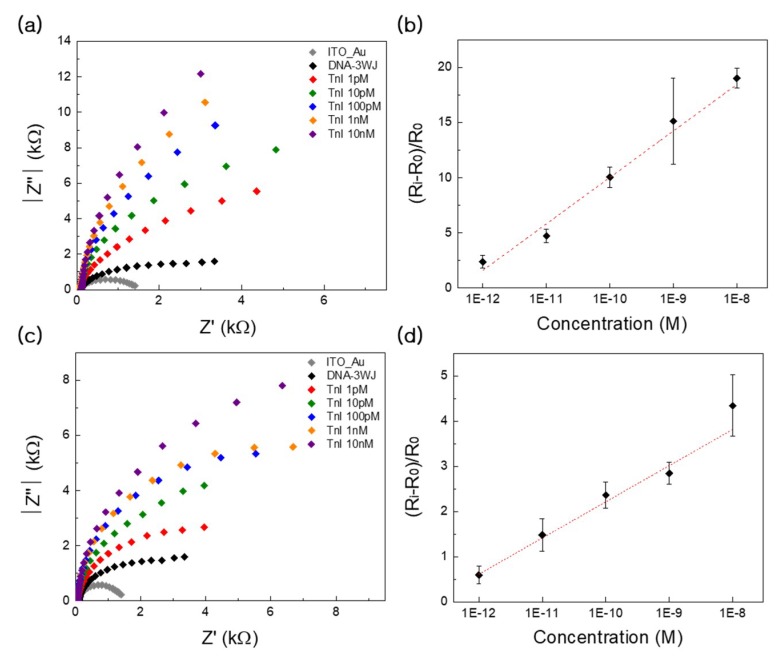
(**a**) Electrochemical impedance spectra of DNA 3WJ/AuNC on ITO substrate with various concentrations of cTnI in dimethylamine buffer (from 1 pM to 10 nM). (**b**) The relationship of (R_i_ − R_o_)R_o_ vs. cTnI concentrations (from 1 pM to 10 nM) in dimethylamine buffer. (**c**) Electrochemical impedance spectra of DNA 3WJ/AuNC on ITO substrate with various concentrations of cTnI in 10% diluted human serum (from 1 pM to 10 nM). (**d**) The relationship of (R_i_ − R_o_)R_o_ vs. cTnI concentrations (from 1 pM to 10 nM) in 10% diluted human serum. R_0_ means the electron transfer resistance of bioprobe-modified electrode measured in pure dimethylamine buffer before detection. Ri indiacted the electron transfer resistance of fully modified electrode measured in dimethylamine buffer containing specific concentration of bioprobe. Error bar represents relative standard deviation of eight independent experiments.

EIS measurements were also performed on the DNA 3WJ/AuNC-modified electrode in the presence of cTnI in a manner identical to that of the LSPR test. Quantitative assessment of sensitivity was performed using serial dilutions of cTnI (1 pM, 10 pM, 100 pM, 1nM, and 10 nM) in dimethylamine buffer. The typical responses of the biosensor equipped with the cTnI measured by EIS are shown in (Figure 5a). The *R*_ct_ measured for the biosensor prior to cTnI detection, as to denoted as *R*_0_ (Figure 5a, black dots), was used for the calculation of relative response towards a specific analyte. Addition of increasing concentrations of cThI increases the *R*_ct_ of the equivalent circuit, and this value is denoted as *R*_i_. The optimal dynamic range exhibiting a quality linear relationship was determined between the target concentration and the bioprobe relative value of R_ct_ ([R_i_ − R_0_]/R_0_) as shown in (Figure 5b). The highest concentration of cTnI in this study caused a significant increase in R_i_ (~20%), and the observed increases in R_i_ linearly varied with the cTnI concentration range of 1.0 pM to 10 nM. For our system that used a dimethylamine buffer, the LOD value was determined as 497 fM in buffer through error bar and slopes of (Figure 5b). This is in good agreement with the basic knowledge that the analysis of *R*_ct_ is an extremely useful detection method that displays a very low detection limit. The quality analytical parameters, such as the linear responses towards the cTnI present in the buffer solutions, suggested that the biosensor could be successfully applied for detection of humoral responses in serum. To confirm this, we conducted a quantitative assessment of the biosensor in 10% diluted human serum. The EIS spectra recorded for electrode-incorporated cTnI in the presence of human serum are presented in (Figure 5c), and the relative *R*_ct_ values are described at a cTnI concentration range of 1.0 pM to 10 nM (Figure 5d). The presence of human serum slightly reduced the impedimetric response of the biosensor towards cTnI; however, the LOD value was obtained as 829 fM in buffer through error bar and slopes of (Figure 5d), indicating that the sensor was able to detect a humoral response in serum at a very low detection limit.

### 3.5. Specificity Tests Using EC and LSPR Methods

**Figure 6:**
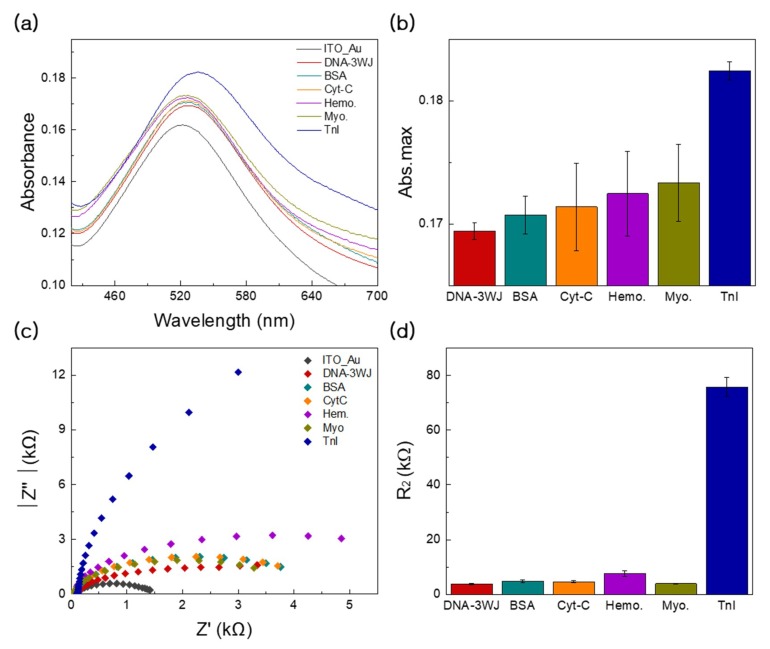
(**a**) Selectivity test of DNA 3WJ/AuNC-based with the various targets including BSA (turquoise line), cytochrome c (yellow line), hemoglobin (purple line), myoglobin (grass line) and cTnI (blue line) through LSPR. (**b**) Change in absorbance peak based on selectivity test with other protein reaction. (**c**) Selectivity test of DNA 3WJ/AuNC-based with the various targets including BSA (turquoise line), cytochrome c (yellow line), hemoglobin (purple line), myoglobin (grass line) and cTnI (blue line) through EIS. (**d**) Change in resistance based on selectivity. Error bar represents relative standard deviation of eight independent experiments.

Figure 6a,b shows the specificity test of the ability of fabricated MF-DNA/AuNC biosensor to detect cTnI (blue color), performed by introducing other proteins such as albumin (green color), cytochrome c (yellow color), hemoglobin (purple color), and myoglobin (grass color) to our LSPR experiment. In (Figure 6a), the UV-VIS spectra result showed little LSPR intensity change compared to that observed after cTnI addition to the fabricated electrode. Additionally, (Figure 6b) displays the absorbance change values with included error bars. Compared to other proteins, the absorbance change of cTnI possessed the lowest SD values; however, when the other proteins were added to the fabricated substrate, greater fluctuations in absorbance change were obtained. 

Figure 6c,d shows the specificity test of the fabricated MF-DNA/AuNC biosensor to detect the cTnI (blue color) in the context of clinical samples, performed by introducing other proteins into diluted serum. These proteins included albumin (green color), cytochrome c (yellow color), hemoglobin (purple color) and myoglobin (grass color) that were used as the controls for EIS experiments. Compared to the other proteins, the addition of cTnI to the fabricated electrode showed the largest relative electron transfer resistance (R_i_ − R_0_/R_0_). This result can be explained by the possibility that only the cTnI specifically reacted with MF-DNA on the AuNC electrode to drastically increase the resistance. Additionally, the detection performance comparison of the fabricated EIS-LSPR dual biosensor and other previously reported biosensors is summarized in (Table 1) [29,30,31]. Thus, the proposed EIS-LSPR biosensor can successfully detect the target cTnI in clinical samples with a high selectivity.

## 4. Conclusions

To rapidly treat AMI patients, the construction of a portable AMI detection system is essential. For the first time, the present study proposed a highly sensitive, label-free cTnI detection biosensor composed of multi-functional DNA on AuNC-modified ITO substrate that functions through a EC-LSPR dual detection method. To incorporate three functionalities into one DNA structure, DNA 3WJ serves as a suitable cTnI biosensor. A cTnI aptamer, a MB group, and a thiol group were sequentially attached to the end of the DNA 3WJ fragment. In particular, the MB acted to simultaneously generate the EC signal and enhance the LSPR signal. The assembled DNA 3WJ also retained multi-functionality. The AuNC-modified ITO substrate was prepared using the electrodeposition method, and the surface morphology was confirmed by FE-SEM. AFM and surface roughness analyses were also used to examine the immobilization process of fabricated biosensor. The LSPR and EIS experiments were performed to confirm the cTnI binding to the DNA 3WJ/AuNC-modified electrode. The LODs for cTnI were validated in both PBS buffer and diluted human serum, respectively. The LOD value was determined to 110 fM in buffer and 840 fM in diluted serum through LSPR experiments, respectively. Also, the LOD value was determined to 497 fM in buffer and 829 fM in diluted serum through EIS experiments, respectively. Based on these results, the proposed EC-LSPR dual detection biosensor composed of multi-functional DNA and AuNC exhibits simplicity, label-free detection, high sensitivity, and selectivity for the clinical assessment of AMI disease. Moreover, the proposed dual detection system can be tuned to sensitivity by introducing the plasmonic nanomaterials such as MoO_3_, MoS_2_ and Bi_2_Se_3_, not only AuNC [32,33,34]. Additionally, the cross testing can reduce the detection error and increase the detection accuracy for portable AMI diagnosis system construction. In the near future, this system can be applied as a portable EC-LSPR dual measurement device that incorporates Arduino and LED-based diagnosis systems for AMI diagnosis.

## Figures and Tables

**Table 1:** Comparison of the fabricated biosensor with other biosensors for troponin I detection in terms of materials, detection method, sensitivity (loss on drying, (LOD)).

Material	Detection Method	Detection Limit	Ref.
Antibody	CV/EIS	5 pg/mL	[29]
Antibody/AuNR	SPR	10 ng/mL	[20]
Antibody	Fluorescence	35 aM	[15]
Antibody	Fluorescence	0.1 pg/mL	[16]
Antibody	SWV	4 pg/mL	[30]
Antibody/GNP	CV	1 ng/mL	[31]
Si-nanowire/Antibody	FET	5 pg/mL	[12]
SnO_2_-nanowire/Antibody	FET	2 ng/mL	[13]
DNA 3WJ/AuNS	CV	24 pg/mL (1 pM)	[14]
MF-DNA/AuNC	EIS/LSPR	110 fM (Buffer, LSPR) 840 fM (Serum, LSPR) 497 fM (Buffer, EIS) 829 fM (Serum, EIS)	Present Work

## Supplementary Materials

The following are available online at https://www.mdpi.com/2079-4991/9/7/1000/s1, Figure S1: The purified cTnIconfirmed by 12% SDS-PAGE. Figure S2: FE-SEM images of AuNC-modified ITO electrode. Figure S3: Cyclic voltamogram of cTnI/DNA-3WJ on AuNC-modified ITO electrode.
