# Supplementary material for: Fabrication of Troponin I Biosensor Composed of Multi-Functional DNA Structure/Au Nanocrystal Using Electrochemical and Localized Surface Plasmon Resonance Dual-Detection Method
Source: Nanomaterials (Basel). 2019 Jul 11;9(7):1000. doi: 10.3390/nano9071000 (PMC6669750; doi:10.3390/nano9071000)
Supplement: Supplementary file 1 [file nanomaterials-09-01000-s001.pdf]

Supplementary Materials

Fabrication of Troponin I Biosensor Composed of Multi-functional DNA Structure/Au Nanocrystal Using Electrochemical and Localized Surface Plasmon Resonance Dual-Detection Method

Optimized Sequence Result of cTnI

(Optimized Sequence Length:645, GC%:57.04)

GGATCC

ATGGCGGATGGCAGCAGCGATGCGGCGCGTGAACCGCGTCCGGCGCCGGCG
CCGATTCGTCGTCGTAGCAGCAATTACCGTGCGTATGCGACCGAACCGCACG
CGAAGAAAAAGAGCAAAATCAGCGCGAGCCGTAAACTGCAGCTGAAAACC
CTGCTGCTGCAGATTGCGAAACAAGAGCTGGAACGTGAGGCGGAGGAACGT
CGTGGTGAGAAAGGTCGTGCGCTGAGCACCCGTTGCCAACCGCTGGAACTG
GCGGGTCTGGGCTTTGCGGAGCTGCAGGACCTGTGCCGTCAACTGCACGCGC
GTGTGGACAAAGTTGATGAGGAACGTTACGACATCGAAGCGAAAGTGACCA
AGAACATCACCGAGATTGCGGACCTGACCCAGAAAATCTTCGATCTGCGTGG
TAAATTTAAGCGTCCGACCCTGCGTCGTGTTCGTATTAGCGCGGATGCGATGA
TGCAGGCGCTGCTGGGCGCGCGTGCGAAGGAAAGCCTGGATCTGCGTGCGC
ACCTGAAACAAGTGAAGAAAGAGGACACCGAAAAGGAGAACCGTGAGGTT
GGTGATTGGCGTAAGAACATTGATGCGCTGAGCGGTATGGAGGGTCGTAAGA
AGAAGTTTGAGAGC

TGAAAGCTT

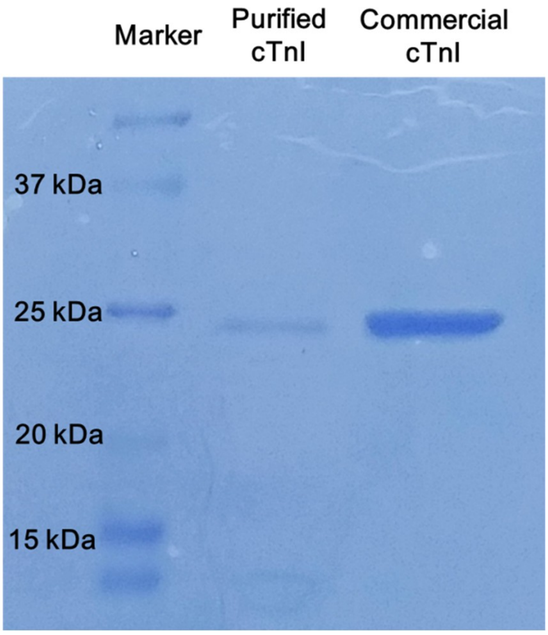

Figure S1. The purified cTnI confirmed by 12% SDS-PAGE.

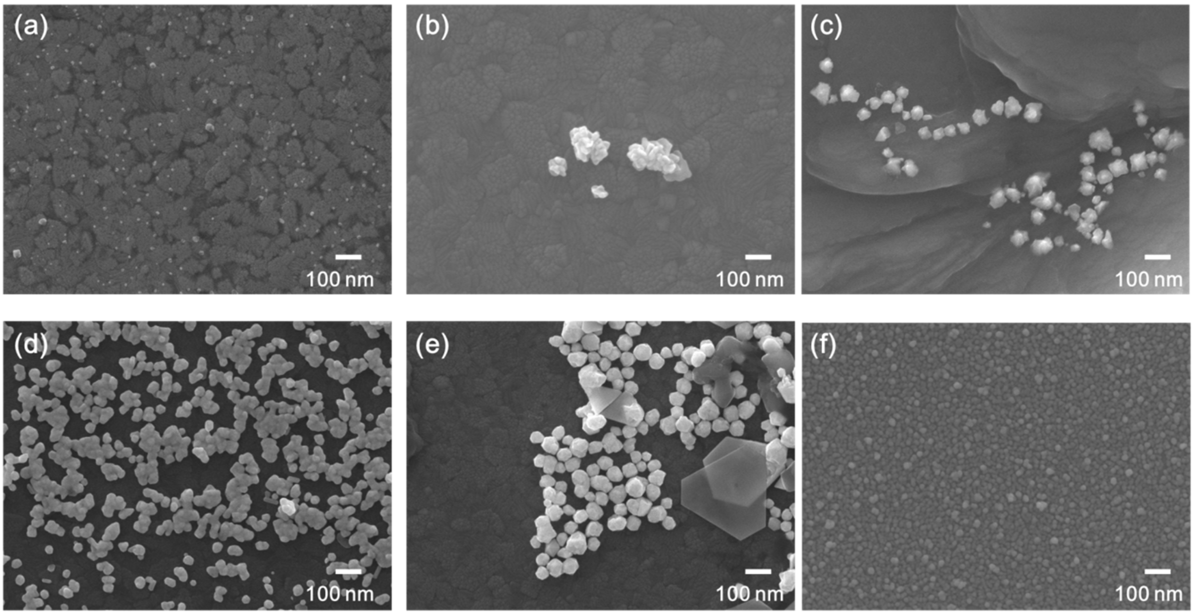

Figure S2. FE-SEM images of AuNC-modified ITO electrode.

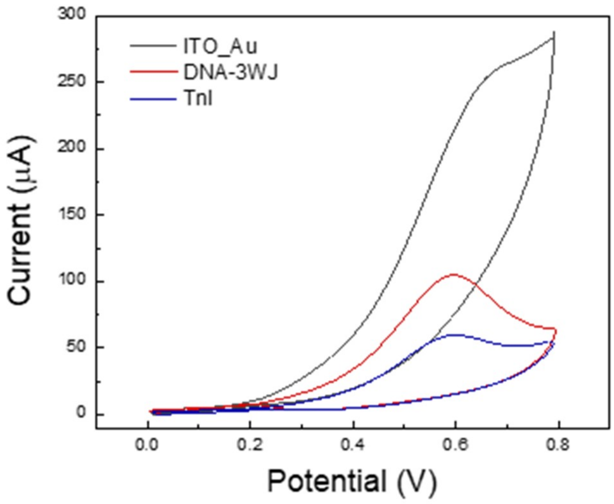

Figure S3. Cyclic voltamogram of cTnI/DNA-3WJ on AuNC-modified ITO electrode.
